# Cookies Fortified with Polyphenols Extracts: Impact on Phenolic Content, Antioxidant Activity, Inhibition of α-Amylase and α-Glucosidase Enzyme, Colour and Sensory Attractiveness

**DOI:** 10.3390/antiox13091108

**Published:** 2024-09-13

**Authors:** Daria Pędziwiatr, Marina Cano Lamadrid, Aneta Wojdyło

**Affiliations:** 1 Department of Fruit, Vegetable and Nutraceutical Plant Technology, Wrocław University of Environmental and Life Sciences, 37 Chełmońskiego Street, 51-630 Wroclaw, Poland; 2 Instituto de Investigación e Innovación Agroalimentaria y Agroambiental (CIAGRO-UMH), Miguel Hernández University, Ctra. Beniel, Km 3.2, 03312 Orihuela, Spain; marina.canol@umh.es

**Keywords:** food fortification, baking products, quince, tilia, pomegranate, passion fruit, sour cherries, haskap, chokeberry, silver skin, rosehip, bioactive potential, α-amylase and α-glucosidase, PCA

## Abstract

The goal of the research was to determine the impact of fortification with polyphenolic compounds on (i) sensory attractiveness (global satisfaction, appearance, colour, odour, flavour, sweetness, bitterness), (ii) content of polyphenols and colour (L*, a*, b*) after the baking process and (iii) their bioactive potential (antioxidants activity and inhibiting of α-amylase and α-glucosidase enzyme). Fortification was made with extracts of polyphenolic compounds of selected plant raw materials rich in polyphenols from quince (fruits), tilia (flowers), pomegranate (skin), passion fruit (endocarp), sour cherries (leaves), haskap and chokeberry (berries), silver skin (coffee beans), rosehip (seeds). Depending on the nature of the polyphenol extract, flavan-3-ols (monomeric and polymeric), phenolic acid, flavonols and anthocyanins were identified in the product in amounts ranging from 53.7 to 212.6 mg/100 g DM. Cookies’ colour (L*, a*, b*) depended on the type of polyphenol extract used for fortification. Cookies with haskap, chokeberry and sour cherry presented the highest antioxidant potential. Cookies with chokeberry, haskap and rosehip presented high activity in inhibiting α-amylase (65.5, 60.6 and 62.2% of inhibition, respectively), but cookies with haskap, silver skin and quince in inhibiting α-glucosidase activity (23.0, 20.4 and 21.4% of inhibition, respectively). In the sensory evaluation, the most attractive were cookies with rosehip and pomegranate (6.3 and 5.8 score, respectively), but the lowest ratings were given to cookies with passion fruit and silver skin but especially quince cookies, which obtained the lowest desirability (3.7 score). The acceptability of fortified cookies was determined to the least extent by monomeric flavan-3-ols and phenolic acids (in minus in odour/flavour, bitterness, sweetness and global satisfaction), but anthocyanins, polymeric procyanidins and flavonols had the most significant positive impact on consumer acceptance of the assessed features, i.e., global satisfaction, odour/flavour, sweetness and bitterness (positive consumer drivers).

## 1. Introduction

A wide range of plant raw materials, including fruits and vegetables, can provide many different nutrients and non-nutritional compounds [1]. Nutrients (including vitamins, proteins, carbohydrates, fats and minerals) are a group of compounds that are necessary for the functioning of the human body, being used as a source of energy, as building material or as ingredients that regulate cellular processes [2]. Non-nutritional compounds (including polyphenols, tri- and tetra-terpenic) are not necessary for the proper functioning of the body, but as they are characterised by the activity of biologically active compounds, they constitute a wide group of compounds that have health benefits that go beyond the basic nutritional value [2].

The high availability of plant products allows for their wide use in nutrition and processing technology, including the production of functional food. Polyphenols that are naturally available in fruits, vegetables, cereals, leaves, roots, coffee and tea are gaining the most attention [3]. Furthermore, waste raw materials (i.e., peel, pulp, seeds or pits) are an excellent source of bioactive substances [4,5]. Both raw materials and pomace resulting from processing and other waste remain a significant source of biologically active compounds (nutritional and non-nutritional). These compounds have significant antioxidant [6,7] and antibacterial properties [8]. Research also confirms the beneficial effect of polyphenols on cardiovascular diseases [9], a key role in the prevention of diseases caused by oxidative stress, as well as a key role in the prevention of cardiovascular diseases and neurological diseases [10].

In the face of many diseases and ailments of the 21st century, ever greater importance is attached to minimally processed food, often fortifying food with protein and fibre [11], vitamins [12] and minerals [13]. An important aspect is also the perception of food and the desire to consume functional food, the main task of which is to provide the body with additional substances, e.g., bioactive ingredients that have a beneficial effect on the human body.

Currently, trends are aimed at preserving or enriching food with functional compounds. The most frequently fortified products are fruit juices, drinks, flour, pasta, bread, salt and confectionery products, including bars and cookies, with fibre, vitamins and minerals, but polyphenol compounds are also increasingly used for this purpose. The addition of elemental iron to wheat flour has allowed this ingredient to be provided in a basic and easily digestible form [14]. Andon et al. [15] prove that calcium absorption improves if the product matrix is liquid (orange and apple juice). Furthermore, enriching orange juice with vitamin D allows the intake of this vitamin to be increased and may have potential beneficial properties in preventing diseases such as diabetes and cancer [12]. Confectionery products, including bars, jellies and cookies, which can also be enriched with bioactive compounds, are becoming an easy and attractive form of snack. So far, cookies have been fortified with fruit pomace, i.e., apple [16], raspberry, chokeberry and blackcurrant [17,18], which improved the quality of the products, their functionality and their dietary fibre content. Research also confirms that the use of pomace in this type of snack with an enriched polyphenol profile allowed the desired product to be produced with an average glycemic index [19]. Research confirmed by Raczkowska et al. [17] also confirms that the addition of fruit pomace to cookies has a positive effect on the content of polyphenol compounds, with antioxidant and antidiabetic effects. It has been proven [20] that the addition of freeze-dried fruit tree leaves rich in polyphenolic compounds increases the content of bioactive compounds in products in direct proportion to their addition. Such products are an alternative for people struggling with diet-related diseases. Orbulescu et al. [21] prove that enriching snack products such as jellies with apple or beetroot juice allows you to obtain a product rich in polyphenol compounds and betacyanins. Plant sources such as quince, tilia, pomegranate, passion fruits, sour cherries, haskap and chokeberry, silver skin and rosehip are typical of the industry in many countries in the world. Additionally, they are typically plant sources rich in polyphenolic compounds represented by different groups such as anthocyanins, flavan-3-ols, phenolic acids or flavonols.

The available literature on the subject has not yet analysed the potential of fortifying confectionery products, i.e., cookies, with polyphenolic compounds isolated from various anatomical parts of plant raw materials (including fruits and berries, flowers, skin, endocarp, leaves, beans and seeds). Therefore, bearing in mind that confectionery products, including cookies, are an attractive form of snack, the aim of this research was to determine the impact of fortification with polyphenolic compounds on (i) sensory attractiveness (global satisfaction, appearance, colour, odour, flavour, sweetness, bitterness), (ii) the content of polyphenols and colour (L*, a*, b*) after the baking process and (iii) their bioactive potential (antioxidants activity and inhibiting of α-amylase and α-glucosidase enzyme). Fortification was made with extracts of polyphenolic compounds of selected plant raw materials rich in polyphenols from quince (fruits), tilia (flowers), pomegranate (skin), passion fruits (endocarp), sour cherries (leaves), haskap and chokeberry (berries), silver skin (coffee beans) and rosehip (seeds). Additionally, the relationship between the tested features was determined, as was which groups of polyphenolic compounds were responsible for the individual sensory properties of fortified cookies.

## 2. Materials and Methods

### 2.1. Preparation of Fortified Cookies

**Scheme 1:**
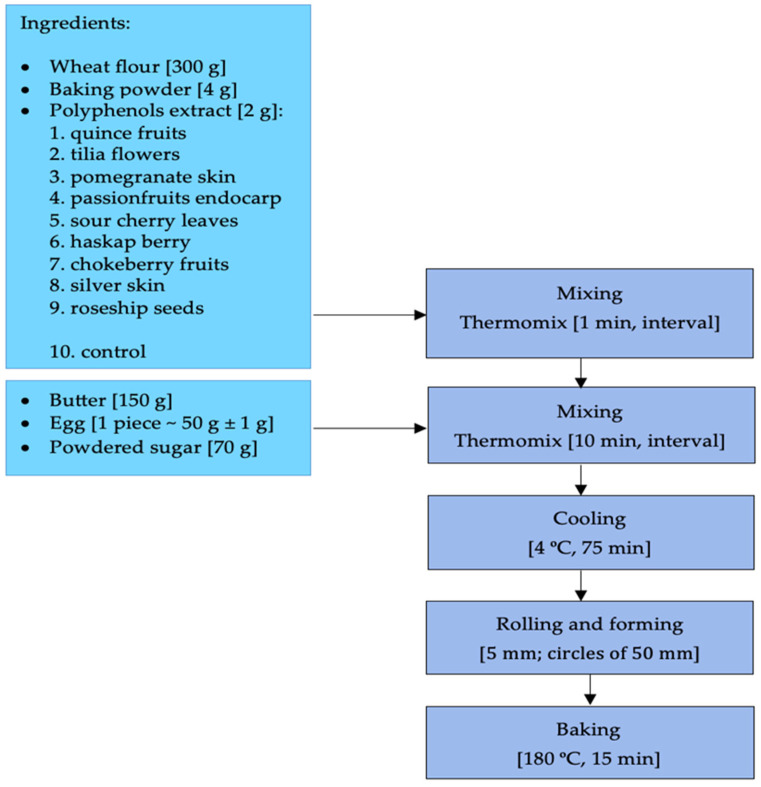
Preparation of cookies fortified with polyphenol extracts.

The cookies are made of flour, butter, powdered sugar, egg, baking powder and salt. The procedure for cookie preparation is shown in Scheme 1.

The cookies were fortified with extracts of polyphenolic compounds of selected plant raw materials:—from quince (fruits), tilia (flowers), pomegranate (skin), passion fruit (endocarp), sour cherries (leaves), haskap and chokeberry (berries), silver skin (coffee beans) and rosehip (seeds)—which were prepared according to the procedure previously described by [22]. Before physical and chemical analysis, the cookies were finely ground in a laboratory grinder (IKA 11A, Staufen, Germany).

### 2.2. Determination of Polyphenolic Compounds

The content of polyphenolic compounds was determined using the Ultra Performance Liquid Chromatography method. The sample (approximately 1 g) was extracted with 5 mL of a mixture containing methanol: H_2_O: ascorbic acid: acetic acid (30:67:2:1, *v*/*v*/*w*/*v*). Extraction was performed twice by incubating the samples for 20 min in an ultrasonic bath (Sonic 6D, Polsonic, Warsaw, Poland) with additional shaking at intervals. The suspension was then centrifuged at 19,000 *g* (MPW-350; Warsaw, Poland) for 10 min, and the resulting supernatant was filtered through a 0.20 μm hydrophilic PTFE membrane (Millex Samplicity Filter, Merck, Darmstadt, Germany) and used for further analysis. Extractions were performed three times.

The quantitative analysis (UPLC-PDA) of polyphenols (flavan-3-ols at 280 nm, flavanols at 360 nm, phenolic acids at 320 nm and anthocyanins at 520 nm) was performed in the manner previously described by Wojdyło, Oszmiański and Bielecki [23] and Wojdyło, Carbonell-Barrachina, Legua and Hernández [24]. Individual polyphenols were separated on an ACQUITY UPLC BEH C18 column (1.7 µm, 2.1 × 100 mm; Waters Corporation, Milford, CT, USA) at 30 °C, where the sample injection was 5 µL. The elution of the sample was completed within 15 min using the gradient and isocratic sequence method at a flow rate of 0.42 mL/min. The mobile phase consisted of solvent A (2% formic acid, *v*/*v*) and solvent B (100% acetonitrile). The program started with a gradient elution with 99–65% solvent A (0–12 min) and then lowering the concentration of solvent A to 0% to condition the column (12.5–13.5 min), after which the gradient returned to the initial composition (99% A). All measurements were repeated three times. The results were expressed in mg/100 g of dry matter (DM).

### 2.3. Analysis of Polymeric Procyanidins by Phloroglucinolysis

The analysis of polymeric fractions of procyanidins was performed using the Ultra Performance Liquid Chromatography method, Acquity Ultra Performance LC Waters. The analysis was performed as previously described by Wojdyło, Oszmiański and Bielecki [23] and Wojdyło, Carbonell-Barrachina, Legua and Hernández [24]. Phloroglucinolysis products were separated on a BEH Shield C18 RP column (1.7 µm, 2.1 × 100 mm; Waters Corporation, Milford, CT, USA) with solvent A (2.5% CH_3_COOH in H_2_O, *v*/*v*) and solvent B (100% acetonitrile). The analysis was performed at 15 °C, where the flow rate was 0.45 mL/min, and the sample injection volume was 5 µL. Fluorescence was recorded at an excitation wavelength of 278 nm and an emission wavelength of 360 nm. Calibration curves for quantification were obtained for procyanidin B2, and (+/−)-(epi)-catechin. The average degree of polymerisation was calculated as the molar ratio of all flavan-3-ol units to the final (+/−)-(epi)catechin units. All samples were analysed in triplicate, and the results were expressed as mg per 100 g of dry matter (DM).

### 2.4. Determination of Antioxidant Activity Using the Method ABTS^o+^, FRAP and ORAC

The antioxidant activity was tested using the ABTS^o+^ method, which allows for the determination of the ability to reduce the ABTS radical, and the FRAP method, in which the Fe^3+^ ion is reduced to Fe^2+^. Samples for analysis were prepared as described by Wojdyło et al. [25]. 7 mL of methanol: H_2_O: HCl (80:20:1, *v*/*v*/*v*) was added to a sample weighing approximately 1 g, then exposed to ultrasound for 15 min. The samples were left for 24 h at 4 °C. After this time, the sonification procedure was repeated. The absorbance of the sample was measured at 734 nm after a reaction lasting 6 min. The FRAP analysis was performed after a 10-min reaction at a wavelength of 593 nm.

In the case of the ORAC test, which involves measuring the fluorescence decrease, the measurement was performed every 5 min at excitation and emission wavelengths of 493 and 515 nm, respectively. All activity measurements are performed in triplicate using a PC UV-2401 spectrophotometer (Shimadzu, Kyoto, Japan). The antioxidant activity of ABTS^o+^, FRAP and ORAC was expressed in mmoL Trolox-Equivalents (TE)/100 g of dry matter (DM).

### 2.5. Inhibition of α-Amylase and α-Glucosidase Activity

The inhibition of α-amylase and α-glucosidase activities was determined according to the procedure previously described by Wojdyło et al. [22,25]. The inhibition of α-amylase activity was assessed by the ability of α-amylase to hydrolyse α-1,4-glycosidic bonds. However, the inhibition of α-glucosidase activity was assessed based on the interaction of α-glucosidase with PNPG (4-nitrophenyl-α-D-glucopyranose). These activities are measured as a result of the reaction and incubation at 37 °C. Absorbance was measured at 600 nm for α-amylase and 405 nm for α-glucosidase. The reference and control samples contained buffer instead of enzymes and acarbose as a reference substance. Results are expressed as % inhibition activity (mg/mL).

### 2.6. Colour Measurement in the CIE L*a*b* System

The colour of the tested samples was determined using a colorquest device (Spectrophotometer CM-700d, Konica Minolta, Kyoto, Japan). The results are presented in the form of three values: L* (colour brightness), a* (red intensity) and b* (yellow intensity). The measurement for each sample was performed in triplicate.

### 2.7. Sensory Evaluation

General guidance for conducting hedonic tests with consumers in a controlled area—Amendment 1 [26]. A sample group of 75 consumers was recruited at UMH (Spain), between 18 and 60 years old. The main requirement for their recruitment was that they regularly consumed cookies. The consumer study was conducted at the UMH facilities in Orihuela, during two sessions of 3 h. In each session, consumers tested only five cookie samples, plus control cookies. The samples were presented according to a balanced incomplete block design (split-plot): all samples were analyzed by the same number of panellists, but not all panellists studied all samples. Each consumer was served one cookie of each formulation coded with 3-digit numbers, together with the questionnaire. Water and unsalted crackers were given to consumers between samples for palate cleansing. Consumers were asked about their overall level of satisfaction using a 9-point hedonic scale (1 = dislike extremely, 5 = neither like nor dislike, and 9 = extremely like), together with questions regarding attributes’ intensity using a Just About Right (JAR) scale. The attributes were: global appearance, colour, cookie odour, cookie flavour, sweetness, bitterness, astringency, hardness, crunchiness and crumbliness.

### 2.8. Statistical Analysis

Statistical analysis was performed using the Statistica package version 15.03 (StatSoft, Kraków, Poland). Significant differences (*p* ≤ 0.05) between means were determined by one-way ANOVA with Tukey’s test. Principal component analysis (PCA) was performed using the XLSTAT statistical software for Microsoft Excel 2017 (Microsoft Corp., Redmond, WA, USA).

## 3. Results and Discussion

### 3.1. Content of Polyphenol Compounds in Fortified Cookies

Polyphenols are one of the most important secondary metabolites produced by plants. They have strong anti-inflammatory and antioxidant properties [27]; hence, it is advisable that they be delivered to the body successively in various foods, also by fortifying food products. The quantitative analysis carried out using UPLC-PDA confirmed the presence of four groups of polyphenolic compounds in the analysed products: flavan-3-ols, phenolic acids, flavonols and anthocyanins. Additionally, polymeric procyanidins belonging to flavan-3-ols were determined. These analyses allowed us to determine which product after the fortification process is characterised by high retention, which is important because biscuit-type products are subjected to high temperatures during the baking process. The dominant group of polyphenolic compounds were flavan-3-ols, including polymeric procyanidins, next phenolic acids, and finally flavonols. However, the least numerous groups represented in two samples (the cookie with haskap berry and the cookie with chokeberry) were anthocyanins. The main reason for significant differences in the content of polyphenol compounds in the tested cookies was the origin and source of the polyphenol preparation used in the fortification process. Analysing the results obtained from Table 1, it was found that the highest total retention of the number of polyphenolic compounds (212.6 mg/100 g DM) was achieved by the addition of the preparation obtained from tilia (flowers). The lowest total amount of polyphenols (64.3 mg/100 g DM) in relation to the control sample was obtained in cookies with passion fruit (endocarp). Cookies with pomegranate and silver skin and cookies with rosehip and quince had similar content. A trace quantity of polyphenolic compounds was determined in the control cookies, which clearly indicates the right direction of activities aimed at fortification in polyphenols.

Flavan-3-ols are substances that have many health-promoting properties, from antibacterial activities, through the protection of the circulatory system against diseases, to anticancer activities [28]. The content of these compounds in the tested cookies ranged from 13.4 (cookie with haskap berry) to 35.2 mg/100 g DM (cookie with silver skin). Fortified cookies with quince (fruits) and pomegranate (skin) had an equally high content (Table 1).

Apart from the monomeric structure ((+/−)-(epi)-catechin), flavanols also occur in plants as procyanidin dimers and polymers. These are compounds that, through the multiplication of hydroxyl groups in various arrangements in their structure, are characterised by strong antioxidant properties, minimizing cell damage caused by oxidative stress, thus demonstrating protective effects in the prevention of some chronic metabolic diseases [29]. They also modulate biological activity and have anti-inflammatory and cardiovascular protection effects [30,31]. The highest content of polymeric procyanidins among fortified products was determined in cookies with tilia (flowers). As also confirmed by the research of Nowicka and Wojdyło [32], tilia flowers are a rich source of polymeric procyanidins. The remaining cookies were characterised by a low content of polymeric procyanidins, below 60 mg/100 g DM. It was also a small amount compared to the control sample, which contained 35.1 mg/100 g DM.

The next group of compounds marked were phenolic acids. These usually occur in plants in the form of esters and glycosides [33]. Their content ranged from 1.12 (cookie with passion fruit) to 29.0 mg/100 g DM (cookie with pomegranate). These compounds were not detected only in cookies non fortified with polyphenol preparations (Table 1). Products containing phenolic acids have anti-inflammatory, neuroprotective and antimicrobial effects [34]. However, the research of Zhao et al. [35] also confirms their antihypertensive properties.

Flavonols are a group of compounds that reduce the risk of cancer formation and development and have antimutagenic effects through their detoxification activity [36]. Their highest content was measured in cookies with sour cherry leaves (52.5 mg/100 g DM) and in cookies with tilia flowers (33.1 mg/100 g DM). In the remaining fortified cookies, the amount of flavonols was determined to be low, below 6.2 mg/100 g DM. These compounds were not determined in two samples (control and cookie with rosehip seeds). Flavonols are more specific in anatomical parts of plants such as leaves, flowers and roots. Research by Wojdyło et al. [37] confirm that both fruits and leaves of common quince are a valuable source of compounds such as phenolic acids.

Due to the specific colour of anthocyanins, these compounds were characteristic of two analysed products, i.e., the cookie with haskap berry and the cookie with chokeberry.

Anthocyanins are colourful, water-soluble compounds found in glycosylated form in fruits, vegetables or, more precisely, in their individual anatomical parts. Twice as many of these compounds were determined in the cookie with haskap berry (Table 1) as in the cookie with chokeberry. This class of flavonoids, as natural pigments, is responsible for the attractive red, purple and blue colours of fruits, berries and vegetables, as well as products made with their participation [38]. Foods rich in anthocyanins, thanks to their strong antioxidant properties, can provide health benefits such as weight control, disease prevention and vision improvement [39,40]. Anthocyanins are compounds that are easily degraded under the influence of temperature [41]. Gąsiorowski et al. [42] confirms that the presence of anthocyanins in chokeberry influences the antimutagenic effect, which is mainly based on their free radical scavenging effect.

### 3.2. Biological Activity

#### 3.2.1. Antioxidant Activity

To assess precisely the potential of antioxidant activity, three different tests, independent in their mechanisms of action, were used: ABTS^o+^, FRAP, ORAC (mmoL TE/100 g DM; Table 1). Antioxidant activity is the ability to remove free radicals and prevent oxidative damage, which is a significant cause of inflammation and the etiopathogenesis of lifestyle diseases [43,44]. The antioxidant activity in the tested samples compared to the control sample ranged from 0.3 to 2.2 mmoL TE/100 g DM and 0.3 to 0.9 mmoL TE/100 g DM for the ABTS^o+^ and FRAP tests, respectively. However, the activity determined by the ORAC test ranged from 1.4 to 3.5 mmoL TE/100 g DM. Fortified cookies with haskap berry were characterised by the strongest activity in all tests. The lowest antioxidant activity was found in cookies with passionfruit endocarp, silver skin and pomegranate skin for the ABTS^o+^, FRAP and ORAC tests, respectively. Raczkowska et al. [17] and Hossain et al. [45] confirm that various types of enrichment in shortcrust products (with blackcurrant pomace) increase their antioxidant capacity. However, research conducted by Dauber et al. [46] indicates the strong antioxidant activity of cookies with silver skin. These benefits are related to the presence of bioactive compounds, mainly caffeine and chlorogenic acid derivatives, which occur in the largest amounts and have high antioxidant potential [47]. Research conducted by Davidov-Pardo et al. [48] showed significantly stronger antioxidant activity when microencapsulated grape seed extract was added to cookies. This form allowed the thermal degradation of the extract itself to be reduced and thus had a more beneficial effect on the analysed antioxidant potential.

#### 3.2.2. Inhibition of α-Amylase and α-Glucosidase

Diabetes is a complex disease, and the incidence of the disease has been increasing continuously in recent years [49]. Additionally, Harborg et al. [50] confirms that diabetes significantly affects the prognosis of other diseases such as breast, prostate or colon cancer. Carbohydrates supplied with food are hydrolysed by pancreatic α-amylase and intestinal α-glucosidase, which are responsible for the breakdown of oligo- and disaccharides into monosaccharides that are easily absorbed in the body [25]. The effectiveness of α-amylase inhibition in the conducted studies ranged from 46.0 (cookie with quince fruits) to 65.5% (cookie with chokeberry). However, the α-glucosidase inhibition efficiency ranged from 6.1 (cookie with sour cherry leaves) to 23.0% (cookie with haskap berry). The control trial, without the addition of polyphenols, presented weak activity in inhibiting enzymes responsible for antidiabetic properties, below 9% inhibition (Table 1). Research conducted by Ramón-Canul et al. [51] proves the beneficial antidiabetic effect of cookies enriched with *Mangifera indica* L. leaves or chokeberry pomace rich in polyphenols and fibre [17]. The inhibition of α-amylase by polyphenols is due to binding interactions between polyphenols and the enzyme. According to the scientific literature data [52,53] polyphenols are one of the main compounds that have an inhibitory effect on α-amylase. The inhibition of α-amylase by these compounds is related to the molecular structure, as this inhibition results from the interaction that forms between polyphenols and the enzyme [54,55,56]. The fortified cookies developed in the study may be a good alternative for people with diabetes or obesity. These are lifestyle diseases, and people affected by them have limited opportunities to eat sweet snacks. The use of extracts in this type of snack could be a perfect diversification of their snack diet.

### 3.3. Colour Analysis

Colour is an important sensory factor shaping the attractiveness and acceptance of products. The colour parameters of the tested cookies are presented in Table 2.

The following values were obtained for the control sample: L* (71.7), a* (4.81), b* (31.8). Cookies with tilia flowers (L* = 69.8) and cookies with quince fruits (L* = 69.5) were brighter than the control cookies. The obtained results showed that cookies with haskap berry (30.5) and cookies with chokeberry (33.2) had the least brightness. It was shown that their colour was the darkest (low values of the L* parameter), with a blue-violet shade (low value of the b* parameter), which is related to the characteristic content of coloured compounds in these cookies, such as anthocyanins and their colour change under the influence of baking temperature. The intensity of the red colour was stronger for cookies with silver skin and cookies with chokeberry and weaker for cookies with tilia flowers and cookies with pomegranate skin. In the study by Canalis et al. [57], cookies enriched with peach pulp showed a darker colour compared to the control sample.

### 3.4. Consumer Rating

**Figure 1:**
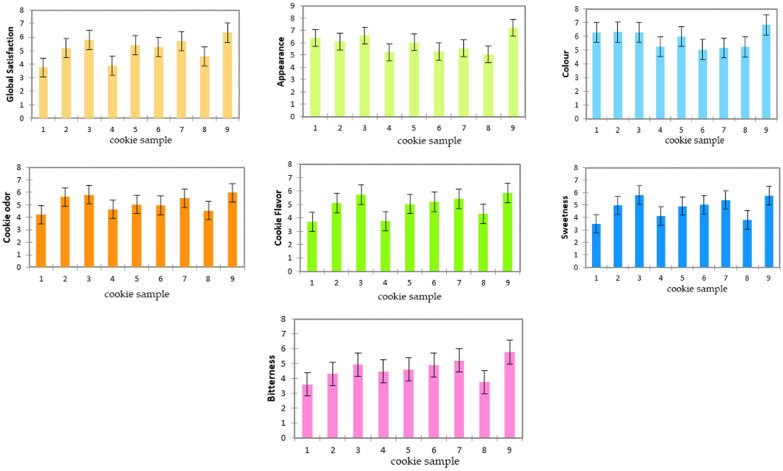
In the following graphs, the consumer acceptance of each attribute is shown (only statistically significant attributes are included). Cookie sample: 1—quince fruits, 2—tilia flowers, 3—pomegranate skin, 4—passionfruits endocarp, 5—sour cherry leaves, 6—haskap berry, 7—chokeberry, 8—silverskin, 9—roseship seeds.

A sensory assessment was carried out using a 9-point hedonic scale to check whether the tested fortified cookies would meet consumer expectations regarding senses and aesthetics—i.e., in terms of overall assessment, appearance, colour, smell, taste, sweetness and bitterness (Figure 1). 

In the overall desirability rating, cookies with rosehip seeds received the highest scores (6.3), followed by cookies with pomegranate skin (5.8). However, cookies with quince fruits received the lowest desirability rating (3.7). In terms of overall appearance, cookies with rosehip seeds (7.2), pomegranate skin (6.6), quince fruits (6.4) and tilia flowers (6.1) were also rated the highest, while the overall appearance of cookies with silver skin (5.0) was rated the lowest. Colour is considered a basic physical property of food, influencing the assessment of external quality [58].

The most acceptable and attractive colour in the cookie evaluation was for cookies with rosehip seeds (6.9), tilia flowers (6.3), quince fruits (6.3) and pomegranate skin (6.3). The lowest scores were given to cookies with haskap berry (5.0) and cookies with chokeberry (5.2), i.e., those in which anthocyanins were the dominant compounds. Anthocyanins are sensitive to environmental, chemical and physical factors, including high temperature, thus losing their attractive colour and red shade. Respondents described their colour as darker than the others, with a shade of purple, hence the lower scores in their colour assessment. Davidov-Pardo et al. [48] indicates that cookies enriched with grape seed extract are tarter in the opinion of consumers and have an aroma and taste like whole grain flour. In the above-mentioned studies [48], consumers rated cookies without and with the extract at a similar level. These studies confirm the importance of information and education about the health-promoting properties of fortified products resulting from their consumption. Due to the growing interest in functional foods, especially bioactive substances, food producers are looking for new sources and carriers of these substances, thus trying to fortify food with them. In the case of two descriptors, namely smell and taste, the highest consumer acceptability was achieved by cookies with rosehip seeds, followed by pomegranate skin, while the lowest was cookies with quince fruits, because the evaluators pointed out their bittersweet aftertaste, which in their opinion was not attractive. The taste of cookies with passionfruit endocarp was also rated relatively low (3.8). Of all the assessed features, the highest scores were for appearance and colour, which indicates that these are the features that determine the purchase of a given product. Górecka et al. [59], using consumer evaluation, proved that the addition of raspberry pomace did not have a negative impact on the organoleptic properties of the product and was accepted by consumers. Analysing the results of assessing the acceptability of cookies’ sweetness, fortified cookies with pomegranate skin (5.8) and cookies with rosehip seeds (5.8) were highly rated.

### 3.5. Principal Components Analysis (PCA) and Hierarchical Cluster Analysis (HCA)

**Figure 2:**
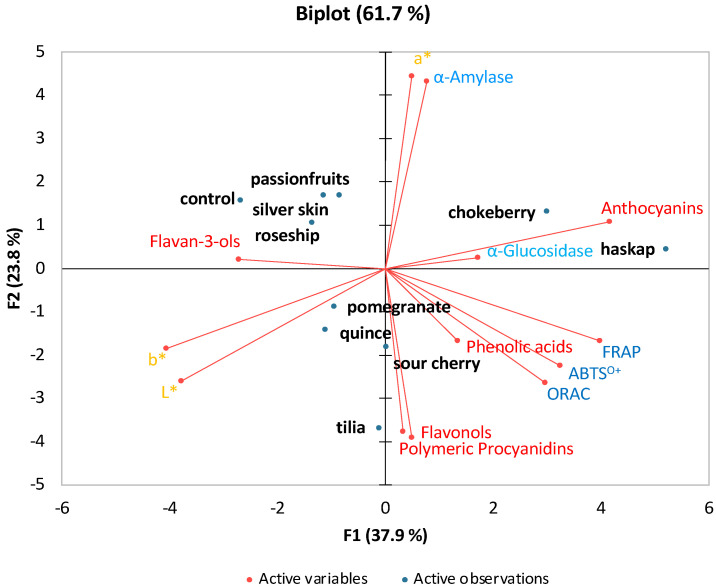
Analysis of the main components (PCA) biplot of fortified cookies and phenolic compounds, ABTS^o+^, FRAP and ORAC activity, antidiabetic activity and parametr L*a*b*.

To illustrate and interpret multidimensional data sets, principal component analysis—PCA—was performed. This analysis considered the following parameters: groups of polyphenolic compounds (flavan-3-ols including procyanidin polymers, phenolic acids, flavonols, anthocyanins), effects of biological activity: antioxidant (ABTS^o+^, FRAP and ORAC), α-amylase inhibitory potential, α-glucosidase and colour indicators (L*, a*, b*) of the tested fortified cookies. The PCA model (Figure 2) presents the most important variables and explains the relationships between the tested fortified biscuit products.

The biplot indicates that 61.7% of the total variance in the data is represented by F1 and F2. Of these two principal components, F1 explains 37.9% of the total variance, and F2 explains 23.8%.

PCA analysis confirms previous conclusions regarding the differential availability of individual groups of polyphenolic compounds in cookies and their contribution to antioxidant and antidiabetic activity. As confirmed by positive F1 values (right half of the graph), cookies with haskap berry and cookies with chokeberry were rich in anthocyanins and showed high antioxidant activity (ABTS^o+^, FRAP, ORAC) and were responsible for high properties to inhibit the activity of enzymes α-amylase and α-glucosidase. The a* parameter was also correlated with these products, resulting from the anthocyanin content in fortified cookie products. Cookies with passion fruit endocarp, silver skin, rosehip seeds, pomegranate skin, quince fruits and control presented a high monomer of flavan-3-ol content and similar lightness (L*) and yellow-green colour shade (b*).

This group of cookies was also characterised by a lower content of polymeric procyanidins and flavanols. PCA analysis also indicates a strong correlation of cookies with tilia flowers with polymeric procyanidins and cookies with sour cherry leaves with flavonols.

**Figure 3:**
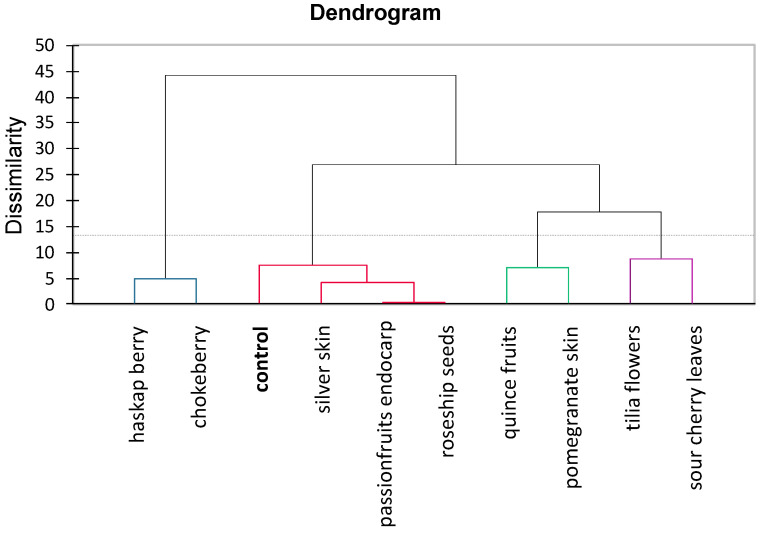
Dendrogram of agglomeration hierarchical clustering (AHC) for fortified cookies. Color line means the same clustering.

Agglomeration and hierarchical clustering were also performed to investigate the relationship between biscuit fortification with polyphenol extracts and biological activity and colour. The AHC dendogram is shown in Figure 3.

The line at 13.2% in the graph represents automatic truncation, showing four homogeneous groups. An early combination of the following cookies—haskap berry and chokeberry, control, rosehip seeds, quince fruits and pomegranate skin, tilia flowers and sour cherry leaves—showedthe greatest similarity of these samples in terms of their analysed polyphenol profile and biological activity and colour. The presented differences confirm the relationships indicated in the PCA.

**Figure 4:**
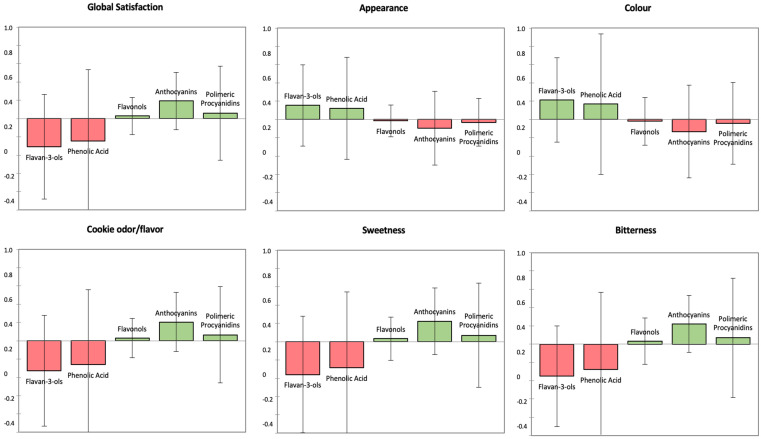
Partial Least Squares Regression (Standardised coefficients. 95% Conf. Interv). Drivers of consumer acceptance of the developed samples. Reddish colour means negative correlation, while greenish colour means positive correlation.

The influence of individual groups of polyphenols as an ingredient shaping sensory properties on its acceptability was also analysed. These relationships are presented in Figure 4. The presented data show that flavan-3-ol and phenolic acid monomers had the greatest negative impact on the acceptance of the assessed sweetness, bitterness and cookie odour/flavour (negative consumer drivers). These relationships also largely determined global satisfaction. However, both flavan-3-ols and phenolic acids monomers had a positive effect on the consumer acceptance of the assessed features (positive consumer drivers), i.e., appearance and colour while flavonols, anthocyanins and polymeric procyanidins had a negative effect on them. It was also observed that among flavonols, anthocyanins and polymeric procyanidins, the content of anthocyanins had the highest positive correlation with global satisfaction, cookie odour/flavour, sweetness and bitterness, being positive consumer drivers.

It is known that flavan-3-ols are the compounds largely responsible for the bitter and astringent sensory properties, which has been repeatedly emphasized in the scientific literature [60,61]. Passoni et al. [62] also confirms that compounds belonging to the flavonol class have a significant impact on the sensory properties in the oral cavity. However, Nishiyama-Hortense et al. [63], based on consumer evaluation, indicate the beneficial effect of anthocyanins on the uniform colour, smell and taste of jelly candy.

## 4. Conclusions

The research confirmed the significant impact of fortification with extracts of polyphenolic compounds in cookies. The tested products contained polyphenolic compounds (from 64.3 to 212.6 mg/100 g DM) belonging to the following groups: flavan-3-ols, flavonols, phenolic acids and anthocyanins. The dominant group occurring in almost all fortified cookies were flavan-3-ols, including procyanidin polymers, > phenolic acids >> flavonols. However, the least numerous group, represented in two samples (cookie with haskap berry as 65.6 mg/100 g DM, and chokeberry as 31.2 mg/100 g DM), was anthocyanins. The highest ABTS^o+^, FRAP, ORAC activity was confirmed for cookies with haskap berry—2.2, 0.9 and 3.5 mmol TE/100 g DM, respectively. Furthermore, the antidiabetic activity was more beneficial in cookies fortified with polyphenols than in the control sample.

Cookies with chokeberry, haskap and roseship presented high α-amylase inhibition (65.5, 60.6 and 62.2% of inhibition, respectively), while cookies with haskap, silver skin and quince demonstrably inhibited α-glucosidase activity (23.0, 20.4 and 21.4% of inhibition, respectively). The potential of these cookies is about 10 times higher than control samples without fortification by polyphenol extracts. The acceptability of fortified cookies was determined to the least extent by monomeric flavan-3-ols and phenolic acids (in minus in odour/flavour, bitterness, sweetness and global satisfaction), but anthocyanins, polymeric procyanidins and flavonols had the most significant positive impact on the consumer acceptance of the assessed features, i.e., global satisfaction, odour/flavour, sweetness and bitterness (positive consumer drivers).

Fortified cookies are a good source of biologically active compounds and provide a number of health benefits. The conducted research indicates that cookies with chokeberry and cookies with haskap berry may function in the diet as a supplementing snack or a snack introducing bioactive compounds into the diet, including in people with diabetes or obesity. Research indicates that this trend should be followed and developed further in order to introduce functional food to the market for all consumers, not only those suffering from lifestyle diseases and ailments

## Figures and Tables

**Table 1:** Phenolic compounds (mg/100 g DM) and biological activity as antioxidant activity (ABTS^o+^, FRAP, ORAC; mmoL TE/100 g DM) and % of inhibition of α-amylase and α-glucosidase activity (% of inhibition) fortified cookies.

Cookie +	Polyphenol Compounds						Antioxidant Activity			Inhibition [%]	
	Flavan-3-ols		Phenolic Acids	Flavonols	Anthocyanins	Total Polyphenols	ABTS^o+^	FRAP	ORAC	α-Amylase	α-Glucosidase
	Monomeric	Polymeric Procyanidins									
quince (fruits)	34.0 ± 1.7 a	44.0 ± 2.2 cd	23.1 ± 1.2 b	2.8 ± 0.1 d	nd c	103.8 c	0.6 ± 0.1 e	0.43 ± 0.0 de	2.4 ± 0.1 cd	46.0 ± 2.3 e	21.4 ± 1.2 ab
tilia (flowers)	21.1 ± 1.1 c	155.2 ± 7.8 a	3.3 ± 0.2 e	33.1 ± 1.7 b	nd c	212.6 a	1.4 ± 0.1 c	0.5 ± 0.0 cd	3.2 ± 0.2 b	46.4 ± 2.3 e	10.5 ± 0.5 de
pomegranate (skin)	nd g	40.5 ± 2.0 c–e	29.0 ± 1.5 a	0.6 ± 0.1 de	nd c	70.1 d	1.7 ± 0.1 b	0.6 ± 0.0 c	1.4 ± 0.1 e	48.5 ± 2.4 de	14.5 ± 0.7 c
passionfruits (endocarp)	24.8 ± 1.2 b	37.1 ± 1.9 de	1.1 ± 0.1 f	1.3 ± 0.1 de	nd c	64.3 de	0.3 ± 0.0 fg	0.3 ± 0.0 f	2.1 ± 0.1 d	61.6 ± 3.1 a–c	8.8 ± 0.4 ef
sour cherry (leaves)	16.4 ± 0.8 de	37.7 ± 1.9 de	12.9 ± 0.6 c	52.5 ± 2.6 a	nd c	119.3 b	0.7 ± 0.0 ef	0.5 ± 0.0 cd	3.5 ± 0.2 ab	58.3 ± 3.7 bc	6.1 ± 0.3 g
haskap (berry)	13.4 ± 0.7 f	41.7 ± 2.1 c–e	7.2 ± 0.4 d	6.2 ± 0.3 c	65.6 ± 3.3 a	134.1 b	2.2 ± 0.2 a	0.9 ± 0.1 a	3.5 ± 0.2 a	60.6 ± 3.0 a–c	23.0 ± 1.2 a
chokeberry (berry)	15.3 ± 0.8 de	59.8 ±3.0 b	14.4 ± 0.7 c	6.2 ± 0.3 c	31.2 ± 1.6 b	126.9 b	1.0 ± 0.1 d	0.8 ± 0.1 b	2.5 ± 0.1 c	65.5 ± 3.3 ab	11.6 ± 0.6 d
silver skin (coffee beans)	35.2 ± 1.8 a	36.1 ± 1.8 de	3.1 ± 0.2 e	nd e	nd c	74.4 d	0.4 ± 0.1 ef	0.3 ± 0.0 f	2.1 ± 0.1 d	56.0 ± 2.8 cd	20.4 ± 1.0 b
roseship (seeds)	nd g	48.5 ± 2.4 c	27.2 ± 1.4 a	nd e	nd c	75.6 d	0.5 ± 0.1 ef	0.4 ± 0.0 ef	2.2 ± 0.1 cd	62.2 ± 3.1 a–c	8.5 ± 0.4 f
control	18.6 ± 0.9 de	35.1 ± 1.8 e	nd g	nd e	nd c	53.7 e	0.0 ± 0.0 g	0.0 ± 0.0 g	0.9 ± 0.0 f	6.7 ± 3.4 a	8.6 ± 4.4 ef

Results are presented as the average of three independent replicates; nd—not detected; a–g values followed by the same letter in the column are not significantly different (*p* < 0.05; Tukey’s test).

**Table 2:** Colour parameters of fortified cookies by polyphenols.

Cookie +	L*	a*	b*	DE*	dL*	da*	db*	EP
quince (fruits)	69.5 ± 0.7 ab	4.0 ± 0.1 de	30.8 ± 0.4 a	2.5	−2.2	−0.8	−1.0	40.5
tilia (flowers)	69.8 ± 0.4 ab	3.7 ± 0.1 e	27.9 ± 0.4 cd	4.5	−1.9	−1.1	−3.9	36.7
pomegranate (skin)	65.5 ± 0.1 cd	3.7 ± 0.1 e	29.3 ± 0.1 b	6.8	−6.3	−1.1	−2.5	40.3
passionfruits (endocarp)	53.2 ± 0.7 e	6.7 ± 0.4 b	17.5 ± 0.1 f	23.5	−18.5	1.9	−14.3	29.9
sour cherry (leaves)	67.5 ± 0.8 bc	4.7 ± 0.1 cd	27.1 ± 0.3 d	6.3	−4.2	−0.1	−4.7	36.9
haskap (berry)	30.5 ± 0.2 g	5.4 ± 0.1 c	−1.5 ± 0.0 h	53.0	−41.2	0.6	−33.3	8.1
chokeberry (berry)	33.2 ± 0.1 f	6.7 ± 0.1 b	0.3 ± 0.1 g	49.8	−38.5	1.9	−31.5	7.7
silver skin (coffee beans)	52.3 ± 0.2 e	7.8 ± 0.1 a	25.4 ± 0.3 e	20.6	−19.4	3.0	−6.4	42.7
roseship (seeds)	64.7 ± 0.4 d	6.6 ± 0.1 b	28.7 ± 0.3 bc	7.8	−7.0	1.8	−3.1	40.5
control	71.7 ± 0.8 a	4.8 ± 0.3 cd	31.8 ± 0.2 a	-	-	-	-	40.8

L*—lightness; a* (>0)—redness; b* (>0) yellowness. Results are presented as the average of three independent replicates; a–h values followed by the same letter in the column are not significantly different (*p* < 0.05; Tukey’s test.

## Data Availability

The data used to support the findings of this study can be made available by the corresponding author upon request.
